# A phase I/II study of weekly nab-paclitaxel plus cisplatin in chemotherapy-naïve patients with advanced non-small-cell lung cancer

**DOI:** 10.1186/s12885-020-6588-y

**Published:** 2020-02-11

**Authors:** Yoshihiro Hattori, Yuko Kono, Shoichi Itoh, Takako Inoue, Yoshiko Urata, Yoshitaka Kawa, Rie Tohnai, Toru Kumagai, Kazumi Nishino, Ryuji Uozumi, Satoshi Morita, Shunichi Negoro, Fumio Imamura, Miyako Satouchi

**Affiliations:** 1 Department of Thoracic Oncology, Hyogo Cancer Center, 13-70 Kitaoji-cho, Akashi-shi, Hyogo 673-8558 Japan; 2 Department of Thoracic Oncology, Osaka International Cancer Institute, Osaka, Japan; 3 Department of Biomedical Statistics and Bioinformatics, Kyoto University Graduate School of Medicine, Kyoto, Japan; 4 Department of Medical Oncology, Takarazuka City Hospital, Takarazuka, Japan

**Keywords:** Nab-paclitaxel, Cisplatin, Non-small-cell lung cancer, Chemotherapy-naïve patients

## Abstract

**Background:**

The aim of this study was to evaluate the efficacy and safety of nab-paclitaxel plus cisplatin in chemotherapy-naïve patients with advanced non-small-cell lung cancer (NSCLC).

**Methods:**

Chemotherapy-naïve patients with advanced NSCLC were eligible. In the phase I dose-escalation cohort (3 + 3 design), patients received nab-paclitaxel (80 or 100 mg/m^2^ given intravenously on days 1, 8 and 15) plus cisplatin (60 or 75 mg/m^2^ given intravenously on day 1) every 4 weeks. The maximum tolerated dose was not reached. Nab-paclitaxel (100 mg/m^2^ given intravenously on days 1, 8 and 15) plus cisplatin (75 mg/m^2^ given intravenously on day 1) every 4 weeks was selected for the phase II cohort. The primary endpoint was the objective response rate (ORR).

**Results:**

Twenty-three patients (phase I, *n* = 6; phase II, *n* = 17) were enrolled, and 22 patients were eligible. The median age was 67.5 years (range 37–75), 90.9% were males, 45.5% had adenocarcinoma and 81.8% had stage IV disease. The ORR was 59.1% (90% confidence interval (CI); 41.8–74.4), and the disease control rate was 86.4% (95% CI; 66.7–95.3). The median progression-free survival was 5.1 months (95% CI; 4.0–6.7), and the median overall survival was 24.2 months (95% CI; 8.4 months to not estimable). The common grade ≥ 3 adverse events were neutropenia (31.8%), leukopenia (27.3%), lung infection (18.2%) and hyponatremia (18.2%). There was one instance of grade 2 interstitial pneumonia and no treatment-related death.

**Conclusions:**

Nab-paclitaxel plus cisplatin was well tolerated and associated with encouraging response outcomes in chemotherapy-naïve patients with advanced NSCLC. Further investigation is warranted.

**Trial registration:**

UMIN Clinical Trials Registry: UMIN000011776; Date of registration: 17 September 2013; Date of enrolment of the first participant to the trial: 23 January 2014.

## Background

It is difficult to cure advanced non-small-cell lung cancer (NSCLC); however, especially in the last few years, the first-line treatment strategies in NSCLC have undergone significant changes. Prior to first-line treatment, mutations in driver oncogenes such as *EGFR* and *ALK* were analyzed, and if they were positive, a molecular targeting agent was selected. For patients in which the driver oncogene is negative or unknown, immune checkpoint inhibitors (ICIs) alone or in combination with cytotoxic drugs, depending on the programmed cell death-ligand 1 (PD-L1) status in the tumor and the tissue type, have been introduced as first-line treatments. However, treatment with cytotoxic drugs remains one of the standard therapies because sometimes it is difficult to use ICIs as the first-line treatment (e.g., in patients with interstitial pneumonia or autoimmune disease).

Nab-paclitaxel is a 130 nm uniform nanoparticle paclitaxel formulation comprised of paclitaxel and human serum albumin. It does not require Cremophor or anhydrous ethanol solvents to formulate, meaning that steroids or antihistamines do not necessarily have to be taken as pretreatments to suppress anaphylactic symptoms. It also allows for quicker administration than solvent-based paclitaxel (sb-paclitaxel). It may also improve the delayed sensory impairment caused by Cremophor [1–3]. The different properties of nab-paclitaxel compared to sb-paclitaxel also point to its better distribution within tumors than sb-paclitaxel (in vitro) [4]. Meanwhile, in terms of toxicity, the incidence of peripheral neuropathy, which is a concern with sb-paclitaxel, was significantly lower with weekly nab-paclitaxel plus carboplatin than with sb-paclitaxel, and it resulted in a shorter time to recovery from grade ≥ 3 neuropathy to grade 1.

It has been reported that sb-paclitaxel plus cisplatin extends median overall survival (mOS) more than etoposide plus cisplatin (9.9 months vs. 7.6 months; *p* = 0.48); as such, it has come to be considered a SOC [5].

Meanwhile, carboplatin, a derivative of cisplatin, does not require large-volume transfusion to prevent renal dysfunction and came into widespread clinical application in the 2000s. As reported by Rosell et al. in a phase III clinical study comparing sb-paclitaxel plus cisplatin to sb-paclitaxel plus carboplatin [6], sb-paclitaxel plus cisplatin was shown to be noninferior in terms of the primary endpoint of response rate (*p* = 0.45), but the mOS was 9.8 months in the sb-paclitaxel plus cisplatin arm (95% confidence interval (CI); 8.2–11 months) as opposed to 8.2 months in the sb-paclitaxel plus carboplatin arm (95% CI; 7.4–9.6 months), indicating that the sb-paclitaxel plus carboplatin arm was significantly inferior (hazard ratio (HR) 1.22; 90% CI 1.06–1.40; *p* = 0.019). The toxicity profile, however, was generally favorable in the sb-paclitaxel plus carboplatin arm, and it is easy to administer; as such, the platinum agent that is usually combined with sb-paclitaxel is carboplatin (more so than cisplatin). Moreover, a Japanese phase II study on sb-paclitaxel plus cisplatin also showed promising results; the response rate was 31% (95% CI; 16–50%), and the mOS was 14.8 months [7]. There have been, however, reports of grade ≥ 3 neuropathy at incidences ranging between 23 and 40% [5], highlighting the strong neurotoxicity issues. As such, the combination has not been used in the real-world clinical setting.

There have been multiple meta-analysis reports of comparisons between cisplatin and carboplatin to date, and they have reported no significant difference in survival [8–10]. The meta-analysis conducted by Ardizzoni et al. featured a cisplatin combination displaying a superior response rate to carboplatin (HR 1.37; 95% CI 1.16–1.61; *p* < 0.001). Moreover, when combined with a 3rd generation anticancer agent, cisplatin allowed for significantly more favorable survival than carboplatin (HR 1.11; 95% CI 1.01–1.21) [10]. Weekly nab-paclitaxel plus carboplatin actually displayed superiority over sb-paclitaxel plus carboplatin in CA031 [11], a multicenter international phase III controlled trial on lung cancer (the response rates were 33 and 25%, respectively; *p* = 0.005), in terms of all-grade sensory neuropathy (47 and 62%, respectively) as well as grade ≥ 3 adverse events (3 and 11%, respectively). The use of FACT-taxane also showed significantly more favorable results in the weekly nab-paclitaxel plus carboplatin arm than in the control arm. Accordingly, the neurotoxicity issue seen with sb-paclitaxel plus cisplatin conceivably could be reduced by switching the sb-paclitaxel to weekly nab-paclitaxel. Weekly nab-paclitaxel plus cisplatin may improve the toxicity profile compared with sb-paclitaxel plus cisplatin and may be a more effective therapy. As such, we planned a phase I/II study of the efficacy and safety of weekly nab-paclitaxel plus cisplatin in advanced NSCLC.

## Methods

### Patient eligibility

Eligible patients had histologically or cytologically confirmed NSCLC, stage IIIB or IV disease (diagnosed according to the seventh edition of the Union for International Cancer Control staging system) or suffered a recurrence after surgery, had no driver oncogenes (e.g., *EGFR*, *ALK*, *ROS1*, or *RET*) and were aged ≥20 years. Patients were also required to have measurable lesions as defined by Response Evaluation Criteria in Solid Tumors (RECIST) version 1.1; an Eastern Cooperative Oncology Group (ECOG) PS of 0 or 1; a life expectancy ≥3 months; and adequate bone marrow, hepatic, and renal function. Patients with interstitial pneumonia or pulmonary fibrosis recognized on computed tomography (CT) scans, uncontrolled pleural effusions or symptomatic brain metastases were deemed to be ineligible.

The protocol was approved by institutional ethical review boards in each of the participating institutes, and all patients provided written informed consent before enrollment. The study was conducted in accordance with the ethical principles in the Declaration of Helsinki. The study has been registered under the University Medical Hospital Information Network (UMIN) Clinical Trials Registry: identifier UMIN 000011776.

### Study design and treatment

**Fig. 1:**
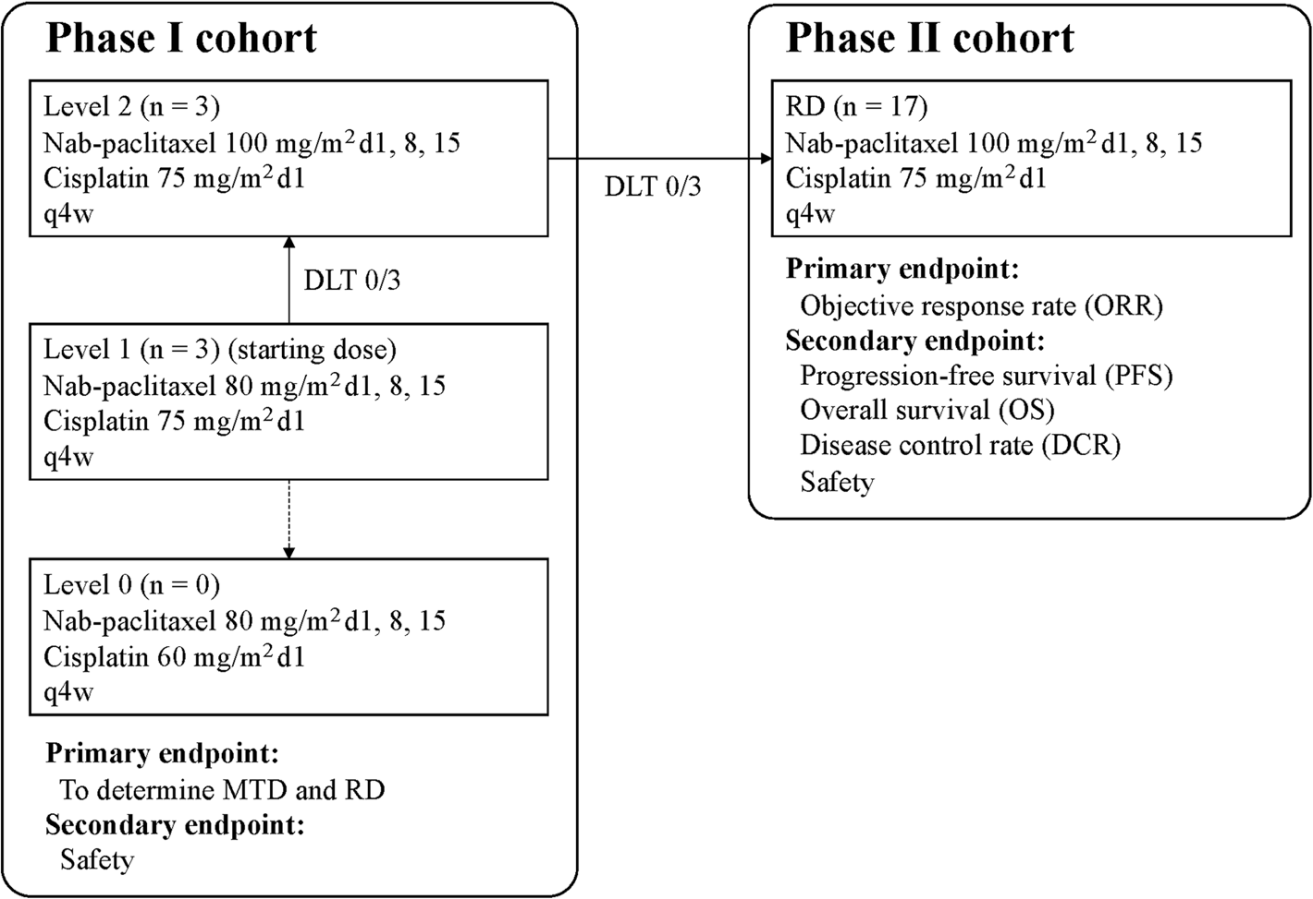
Study design and treatment

The study was designed as a prospective, multicenter, single-arm phase I/II study of first-line combination therapy with nab-paclitaxel and cisplatin. The recommended dose (RD) was determined in phase I, and the efficacy and safety were assessed in phase II. Study design and treatment are presented in Fig. 1. The primary endpoint was treatment efficacy measured as the objective response rate (ORR) in patients who had received at least one cycle of the initial combination therapy. Disease control rate (DCR), overall survival (OS), progression-free survival (PFS), and adverse events (AEs) were also evaluated as secondary endpoints.

Patients received cisplatin at a dose of 60–75 mg/m^2^ given intravenously on day 1 and nab-paclitaxel at a dose of 80–100 mg/m^2^ given intravenously on days 1, 8, and 15. The combination therapy was repeated every 28 days for up to six cycles unless there was evidence of PD or intolerance of the study treatment. Subsequent cycles of treatment were withheld until the following criteria were satisfied: leucocyte count ≥3000/μL; neutrophil count ≥1500/μL; platelet count ≥100,000/μL; PS 0–1; aspartate aminotransferase ≤3.0 × upper limit of normal (ULN); alanine aminotransferase ≤3.0 × ULN; serum total bilirubin ≤1.5 × ULN; serum creatinine ≤1.2 mg/dL; grade ≤ 2 nonhematological toxicities; grade ≤ 2 peripheral neuropathy; and no infection with fever of at least 38 °C.

### Dose-escalation scheme

In phase I, treatment was started at level 1 (cisplatin 75 mg/m^2^, nab-paclitaxel 80 mg/m^2^ q4w). Three patients were initially treated, and if dose-limiting toxicity (DLT) was not observed, we decided to move to level 2 (cisplatin 75 mg/m^2^, nab-paclitaxel 100 mg/m^2^ q4w). If DLT was observed in 1/3 patients at level 1, 3 patients were added. If DLT was observed in 2/6 patients or less, we moved to level 2. If DLT was observed in 3/6 patients or more, we considered level 1 as the MTD, and we decided to examine level 0 (cisplatin 60 mg/m^2^, nab-paclitaxel 80 mg/m^2^ q4w). If DLT was observed in 0/3 patients at level 2, we considered level 2 as the RD. If DLT was observed in 1/3 patients at level 1, 3 patients were added. If DLT was observed in 2/6 patients or less, we considered level 2 as the RD. If DLT was observed in 3/6 patients or more, we considered level 2 as the MTD and level 1 as the RD.

DLT was evaluated by the National Cancer Institute Common Terminology Criteria (NCI-CTC) for Adverse Events version 4.0 and was defined as any of the following during the first cycle of the protocol treatment at each level: leukopenia of grade 4 that lasted more than 4 days, febrile neutropenia, thrombocytopenia of grade 4 or requirement of platelet transfusion, nonhematological toxicity of grade 3 or higher (without nausea, vomiting, anorexia, constipation, and electrolyte abnormalities), and no administration of nab-paclitaxel on days 8 and 15.

### Baseline and treatment assessments

The baseline evaluations included medical history, physical examination, electrocardiogram, ECOG performance status, and laboratory analyses. CT scans of the chest and the upper abdomen, magnetic resonance imaging (MRI) studies or CT scans of the brain, and bone scintigraphy or positron emission tomography (PET)-CT studies were performed for tumor assessment within 28 days of initiation of the study treatment. Tumor measurements were assessed with chest X-ray, CT scans, MRI studies, or bone scintigraphy or PET-CT studies. CT scans were repeated every 2 cycles until PD, MRI studies or CT scans of the brain were repeated every 3 months or upon the appearance of any neurologic symptoms, and bone scintigraphy or PET-CT studies were performed every 6 months or upon the appearance of any bone-related symptoms. Objective tumor responses were based on RECIST version 1.1. The ORR was confirmed via extramural review. Toxicity evaluations were based on NCI-CTC version 4.0.

### Statistical analysis

Analyses of efficacy and safety were based on a December 2017 database lock. On the basis of previous studies [7], we assumed that an ORR of 40% in eligible patients would indicate potential usefulness, while an ORR of 20% would be the lower limit of interest. The number of patients needed to provide 80% power with a one-sided significance level of 5% was calculated to be 32. Taking ineligible patients into account, we intended to enroll 35 patients. Efficacy and safety analyses were planned for patients who received at least one dose of the treatment. Efficacy analysis included patients enrolled in phase I and phase II. The ORR was defined as the proportion of patients achieving the best response of complete response (CR) or partial response (PR) and summarized by a binomial response rate. The two-sided 90% CI, which corresponded to a one-sided significance level of 0.10, was presented using the Wilson method [12] with respect to the efficacy primary endpoint of ORR. The DCR was defined as the proportion of patients achieving CR/PR or stable disease (SD), summarized by a binomial response rate, and assessed with the corresponding 95% CIs. PFS and OS were analyzed using the Kaplan-Meier method to estimate the median values with the corresponding 95% CIs using the Brookmeyer-Crowley method [13]. All analyses were performed using SAS version 9.4 (SAS Institute, Cary, NC, USA).

## Results

### Dose escalation

First, 3 patients were treated with level 1, and DLT was not observed. Next, we moved to level 2, and 3 patients were treated, but DLT was not observed. Therefore, level 2 was decided as the RD.

### Patient characteristics and treatment

Twenty-three patients (phase I cohort, *n* = 6; phase II cohort, *n* = 17) were enrolled from October 2013 to September 2017. The study was terminated before the targeted initial sample size was met because of poor accrual. One patient was excluded from the analysis because of disease progression before the protocol treatment was started. After excluding 1 ineligible patient, 22 patients were ultimately evaluated. The baseline characteristics are shown in Table 1. The median age was 67.5 years (range, 37–75 years), 10 patients (45.5%) had adenocarcinoma histology, 18 patients (81.8%) had stage IV disease, and all patients (100%) were smokers. The median number of treatment cycles was 4 (range, 1–6).

**Table 1:** Patient characteristics

Characteristics		*n* = 22
Age	Median (range)	67.5 (37–75)
Sex, *n* (%)	Male	20 (90.9)
	Female	2 (9.1)
ECOG PS, *n* (%)	0	4 (18.2)
	1	18 (81.8)
Smoking history, *n* (%)	Never	0 (0.0)
	Ever/current	22 (100)
Histology, *n* (%)	Adenocarcinoma	10 (45.5)
	Squamous cell carcinoma	7 (31.8)
	NSCLC, NOS	5 (22.7)
Stage (UICC7), *n* (%)	IIIB	4 (18.2)
	IV	18 (81.8)
	Postoperative recurrence	0 (0.0)
EGFR mutation, *n* (%)	Negative	21 (95.5)
	Unknown	1 (4.5)
ALK transfusion, *n* (%)	Negative	20 (90.9)
	Unknown	2 (9.1)

### Efficacy

**Fig. 2:**
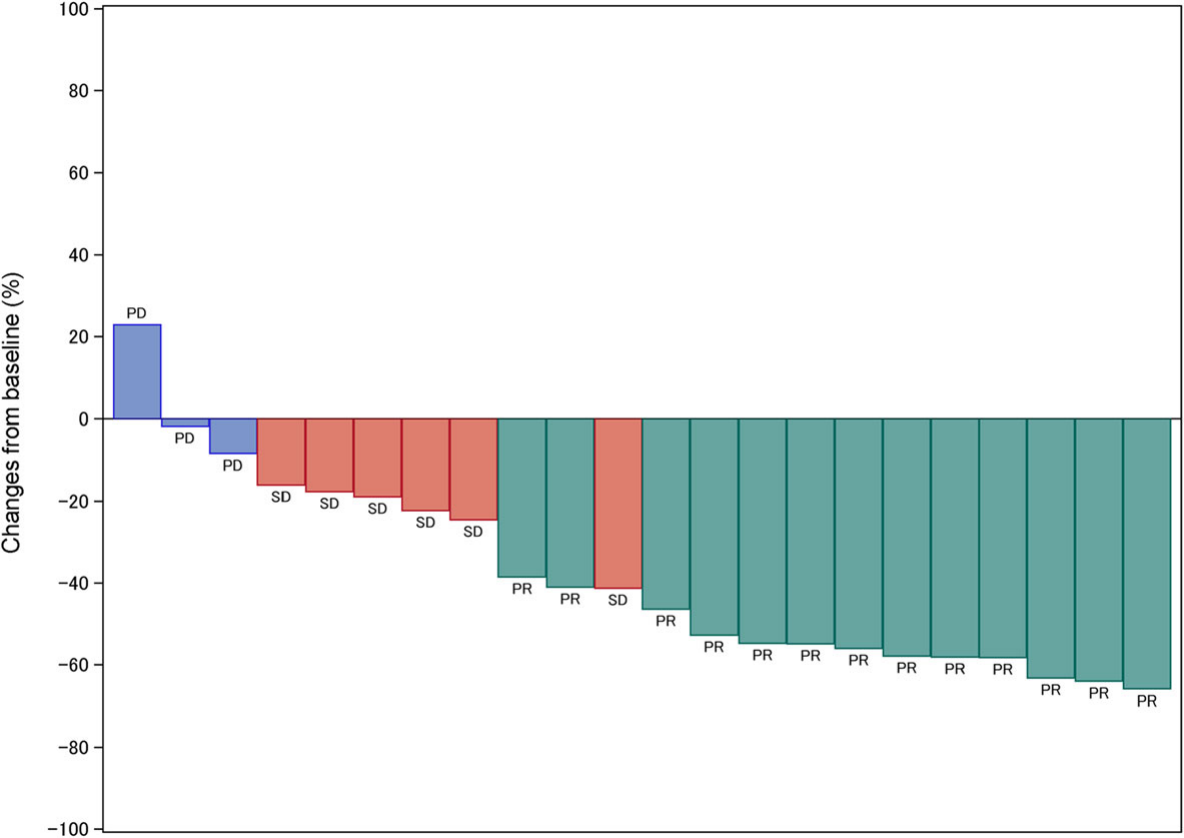
Waterfall plot of best change from baseline

**Fig. 3:**
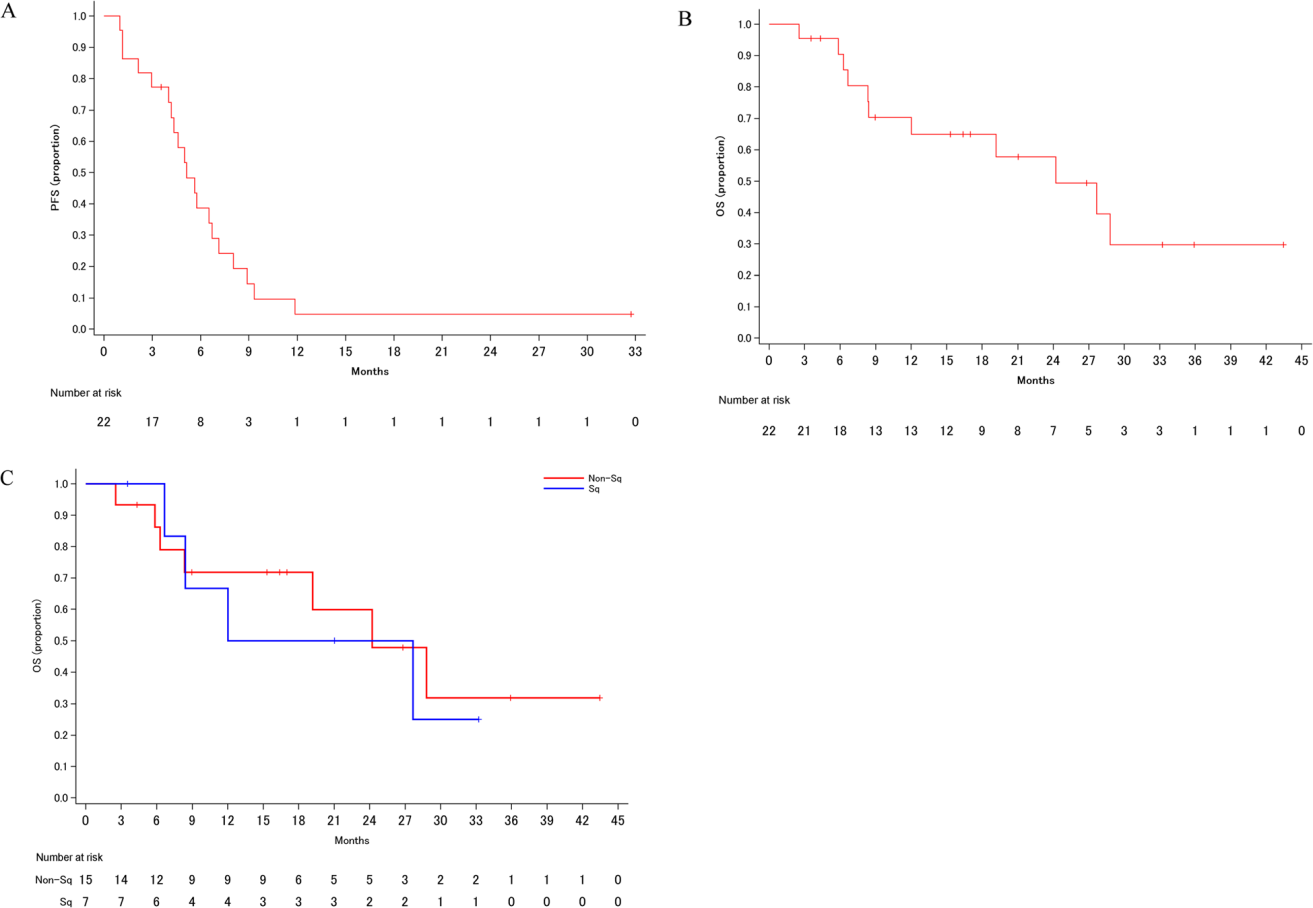
**a** Kaplan-Meier curve for progression-free survival. **b** Kaplan-Meier curve for overall survival. **c** Kaplan-Meier curve for overall survival by histological subtype

Tumor responses are shown in Table 2. The ORR was 59.1% (90% CI; 41.8–74.4%), and the DCR was 86.4% (95% CI; 66.7–95.3%). The maximum changes in tumor measurements are presented in Fig. 2. The median PFS was 5.1 months (95% CI; 4.0–6.7 months) (Fig. 3a), and the median OS was 24.2 months (95% CI; 8.4 months to not estimable (NE)) (Fig. 3b). The OS was 19.8 months (95% CI; 6.7 months to NE) in squamous cell lung cancer (SqCC) and 24.2 months (95% CI; 6.3 months to NE) in non-SqCC (Fig. 3c).

**Table 2:** Tumor response in evaluable patients according to RECIST

	*n* = 22
Objective response	
Complete response, *n* (%)	0 (0.0)
Partial response, *n* (%)	13 (59.1)
Stable disease, *n* (%)	6 (27.3)
Progressive disease, *n* (%)	3 (13.6)
Not evaluable, *n* (%)	0 (0.0)
Objective response rate (%)	13 (59.1)
90% CI, %	41.8–74.4
Disease control rate (%)	19 (86.4)
95% CI, %	66.7–95.3

### Toxicity

Twenty-two patients who received the study treatment were deemed eligible for safety analysis. Hematologic AEs and nonhematologic AEs that occurred in ≥10% of patients are shown in Table 3. The grade 3/4 hematologic AEs were neutropenia (31.8%), leukopenia (27.3%), anemia, and febrile neutropenia (4.5% each). The grade 3/4 nonhematologic AEs were hyponatremia and lung infection (18.2% each) and anorexia, fatigue, hyperkalemia, increased serum amylase, increased lipase, seizure, glaucoma, retinopathy, and hypoxia (4.5% each). There was one instance of grade 2 interstitial pneumonia, and there was no treatment-related death.

**Table 3:** Treatment-related adverse events (*n* = 22)

n (%)	All grade	Grade 3/4
Hematologic adverse events		
Leukopenia	19 (86.4)	6 (27.3)
Neutropenia	20 (90.9)	7 (31.8)
Anemia	22 (100.0)	1 (4.5)
Thrombocytopenia	5 (22.7)	0 (0)
Febrile neutropenia	1 (4.5)	1 (4.5)
Nonhematologic adverse events (≥ 10%)		
Nausea	18 (81.8)	0 (0)
Vomiting	4 (18.2)	0 (0)
Constipation	19 (86.4)	0 (0)
Diarrhea	5 (22.7)	0 (0)
Stomatitis	5 (22.7)	0 (0)
Anorexia	20 (90.9)	1 (4.5)
Fatigue	15 (68.2)	1 (4.5)
Pain	6 (27.3)	0 (0)
Fever	3 (13.6)	0 (0)
Weight loss	3 (13.6)	0 (0)
Alopecia	15 (68.2)	0 (0)
Rash	6 (27.3)	0 (0)
AST increase	4 (18.2)	0 (0)
ALT increase	9 (40.9)	0 (0)
ALP increase	11 (50.0)	0 (0)
Blood bilirubin increase	4 (18.2)	0 (0)
Creatinine increase	7 (31.8)	0 (0)
Hypoalbuminemia	18 (81.8)	0 (0)
Hyperkalemia	15 (68.2)	1 (4.5)
Hypocalcemia	4 (18.2)	0 (0)
Hypokalemia	5 (22.7)	0 (0)
Hyponatremia	5 (22.7)	4 (18.2)
Peripheral sensory neuropathy	6 (27.3)	1 (4.5)
Myalgia	3 (13.6)	0 (0)
Lung infection	4 (18.2)	4 (18.2)

*AST* aspartate aminotransferase, *ALT* alanine aminotransferase

## Discussion

We conducted a phase I/II multicenter joint clinical study on weekly nab-paclitaxel plus cisplatin in treatment-naïve advanced NSCLC patients. The issue, however, was the rapid development of ICIs during the study, including robust development of treatment with combinations of ICIs and chemotherapy, which resulted in slow accrual and eventual completion of the study with a smaller sample than originally planned.

The primary endpoint, ORR, was 59.1%, which was higher than that observed in the CA031 study (33%) and its Japanese subset (35%). While weekly nab-paclitaxel plus carboplatin has displayed a more favorable response rate in SqCC (41% in the CA031 study and 50% in its Japanese subset), the proportion of SqCC in this study (31.8%) was not so high as compared to the CA031 study (44%) and its Japanese subset (14%).

The median PFS (mPFS) was 5.1 months, as opposed to 6.3 and 6.9 months in CA031 and the Japanese subset, respectively, indicating an inferior mPFS. The study enrolled a small number of patients, and the confidence interval was relatively large, making it difficult to determine the quality of these outcomes. It is conceivable that the fact that our study established a maximum of 6 cycles, whereas CA031 did not have a set maximum in place, could have had an influence on the outcomes. Meanwhile, considering that the OS in our study was relatively favorable, weekly nab-paclitaxel plus cisplatin most likely had a minimal impact on subsequent treatments.

Pemetrexed maintenance is one of the standard regimens for first-line treatment of nonsquamous NSCLC. The OS of nonsquamous NSCLC in this study seems to be comparable to the that found in the PARAMOUNT trial [14]. However, it is a limitation that the sample size of our study was small.

No particularly new toxicities were observed above and beyond those seen in other studies to date. Analysis of the Japanese subset in CA031 showed a delay in the initiation of a subsequent course of treatment in 64.3% of the patients, and the median delay was 8 days. Based on these findings, we administered nab-paclitaxel on days 1, 8, and 15, skipping 1 week, in a 4-week cycle. We feel that this maintained the dose intensity that was seen in CA031. Due to the altered schedule, the incidence of all-grade hematological toxicity was virtually the same as that in previous reports. The incidence of grade 3/4 neutropenia was slightly higher (31.8%) than that in CA031 but lower than that in the Japanese subset of CA031 (69% of ≥ Grade 3). In general, myelosuppression in Japanese patients tends to be strong, but it is considered acceptable. There was no hematological toxicity, as in CA031, which frequently requires dose delays and reductions, which facilitated handling. In regard to nonhematological toxicity, there was no particular increase in sensory peripheral neuropathy compared with CA031. While hyponatremia was observed in a relatively large percent of patients, it was adequately manageable. Additionally, while grade ≥ 3 lung infections were observed in 4 patients (18.2%), they all improved. Although grade 2 ILD was observed in 1 patient, there were no treatment-related deaths; safety was within the permissible range.

In recent years, combination therapies consisting of platinum-based combination therapy and ICIs have been developed. In fact, the outcomes from the KEYNOTE-407 trial showed that pembrolizumab added to weekly nab-paclitaxel plus carboplatin prolonged survival [15] and improved health-related quality of life (HRQoL) [16], and it is being introduced into the clinical setting. Recently, the results of the IMpower130 trial also demonstrated the efficacy of the nab-paclitaxel regimen in nonsquamous NSCLC, which has also been introduced into the clinical setting [17]. Moreover, the efficacy and safety of nab-paclitaxel plus carboplatin have been reported in patients with NSCLC with interstitial lung disease [18]. Given that the combination of nab-paclitaxel and platinum salts has been reported to be effective, the results of our study are promising for the future treatment of NSCLC. With reports stating that the combination of nab-paclitaxel and platinum is effective, the results of our study may be promising in the future treatment of NSCLC.

Myelosuppression was mild, and the response rate was promising. As such, weekly nab-paclitaxel plus cisplatin with few treatment delays/skipped treatments (which rarely require schedule changes) could conceivably be a promising candidate for combination therapy with anti-PD-1/PD-L1 antibodies.

## Conclusions

Accordingly, weekly nab-paclitaxel plus cisplatin could be an easy-to-handle treatment option that is highly efficacious with a low incidence of hematological toxicity. Since weekly nab-paclitaxel plus cisplatin is considered promising as a base regimen in combination with ICIs, we would like to conduct a clinical trial of anti-PD-1/−PD-L1 antibodies combined with weekly nab-paclitaxel plus cisplatin for NSCLC in the future.

## Data Availability

The datasets used and/or analyzed during the current study are available from the corresponding author on reasonable request.

## Abbreviations

AE: Adverse event
CI: Confidence interval
CR: Complete response
CT: Computed tomography
DCR: Disease control rate
DLT: Dose-limiting toxicity
ECOG: Eastern Cooperative Oncology Group
HR: Hazard ratio
HRQoL: Health-related quality of life
ICI: Immune checkpoint inhibitor
ILD: Interstitial lung disease
MRI: Magnetic resonance imaging
NCI-CTC: National Cancer Institute Common Terminology Criteria
NSCLC: Non-small-cell lung cancer
ORR: Objective response rate
OS: overall survival
PD: Progressive disease
PD-1: Programmed cell death-1
PD-L1: Programmed cell death-ligand 1
PET: Positron emission tomography
PFS: Progression-free survival
PR: Partial response
PS: Performance status
RD: Recommended dose
RECIST: Response Evaluation Criteria in Solid Tumors
SD: Stable disease
SOC: Standard of care
SqCC: Squamous cell lung cancer
ULN: Upper limit of normal
UMIN: University Medical Hospital Information Network
